# Freshwater Snails at the Biodiversity–Climate–Health Nexus: A Call to Recognize Neglected Models for Eco‐Evolutionary and One Health Research

**DOI:** 10.1002/ece3.73113

**Published:** 2026-02-13

**Authors:** Elodie Chapuis

**Affiliations:** ^1^ MIVEGEC Univ. Montpellier, CNRS, IRD Montpellier France

## Abstract

Freshwater ecosystems are central but overlooked in frameworks addressing the biodiversity‐climate‐health nexus. Among their inhabitants, freshwater snails occupy a unique position at the intersection of ecology, evolution, and disease. They are both sentinels and mediators of environmental change—sensitive to climatic fluctuations, pollutants, and habitat degradation—while serving as intermediate hosts of major zoonotic parasites such as *Fasciola* and *Schistosoma* spp. Their amazing ecological plasticity, diverse reproductive systems, and capacity for rapid adaptation make them powerful yet under‐used models to explore how multiple stressors shape biodiversity dynamics, host‐parasite interactions, and disease risk. Here, I advocate for the recognition of freshwater snails as integrative model systems linking eco‐evolutionary processes with epidemiological outcomes under global change. Their study can reveal general principles on adaptation to multi‐stressor environments, community assembly, and vector competence evolution—core questions in both ecology and One Health. I propose a conceptual framework situating freshwater snails at the biodiversity‐climate‐health nexus to stimulate interdisciplinary research bridging evolutionary ecology, epidemiology, and freshwater conservation. Recognizing these neglected organisms as “nexus sentinels” can advance our understanding of how global change reshapes ecological and health outcomes across aquatic ecosystems.

## Graphical abstract

Freshwater snails occupy a unique position at the intersection of biodiversity, climate, and health. I argue that their ecological plasticity, reproductive diversity, and role as parasite hosts make them powerful yet overlooked model systems for integrating eco‐evolutionary dynamics with One Health challenges. Recognizing snails as “nexus sentinels” can improve our ability to predict and manage disease and biodiversity outcomes under global change.

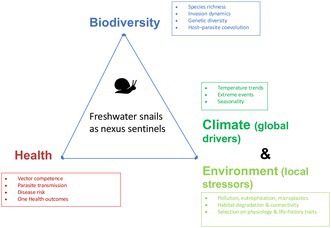

## 1 Freshwater Snails: Forgotten Models in Eco‐Evolutionary and Health Research

Here, I do not aim to provide an exhaustive review, but rather a conceptual synthesis highlighting how freshwater snails can serve as integrative model systems across the biodiversity–climate–health nexus. Freshwater ecosystems occupy less than 1% of Earth's surface yet host a disproportionate share of global biodiversity and deliver critical ecosystem services (Dudgeon 2006; Albert et al. 2021). Recent syntheses further highlight how biodiversity loss in freshwater wetlands undermines functional stability and ecosystem services, reinforcing concerns about the ongoing freshwater biodiversity crisis (Song et al. 2024). These systems are also among the most threatened: habitat loss, pollution, eutrophication, biological invasions, and climate change drive declines faster than in terrestrial or marine environments (Reid 2019). Beyond their ecological importance, freshwater habitats are key systems where biodiversity, climate, and health intersect, providing breeding grounds for pathogens, vectors, and hosts that influence both wildlife and human well‐being (Johnson and Paull 2011; Rohr 2019). The growing integration of zoonotic disease emergence into conservation agendas further underscores that freshwater‐associated pathogens are increasingly recognized as a core challenge for both biodiversity conservation and public health (Smith 2022).

Despite this central role, freshwater systems remain underrepresented in conceptual and empirical frameworks addressing global change. Most research on the integration of ecology, evolution, and health has focused on vertebrate or insect vectors—such as mosquitoes, ticks, or rodents—within well‐developed eco‐evolutionary and One Health frameworks (e.g., mosquito‐borne diseases, rodent‐borne zoonoses, or tick‐borne infections). In contrast, freshwater invertebrates, particularly snails, remain largely overlooked in these integrative approaches despite their central role in the transmission of major parasitic diseases.

This neglect is paradoxical. Freshwater snails are among the most widely distributed invertebrates on Earth, inhabiting nearly all freshwater habitats from tropical wetlands to temperate ponds (Brown 1994). They occupy multiple trophic roles—as grazers, detritivores, and prey for higher consumers—and contribute to nutrient cycling and water quality (Covich et al. 1999; Howard and Cuffey 2006; Flood et al. 2020). At the same time, they are key intermediate hosts of zoonotic parasites, including *Fasciola hepatica* and *Schistosoma* spp., responsible for neglected tropical diseases affecting humans and livestock (Brown 1994; Toledo and Fried 2009; Alba et al. 2021). Their distribution and infection status directly link environmental change, biodiversity—through variation in snail species composition, community structure, and host–parasite diversity—and health outcomes.

Freshwater snails also exhibit remarkable diversity in reproductive systems, ranging from strict self‐fertilization to obligate outcrossing, and in some lineages including parthenogenetic reproduction, sometimes within the same genus (Jarne and Charlesworth 1993; Escobar et al. 2008; Noël et al. 2016). Many species have short generation times and high fecundity, enabling experimental evolution studies impossible in vertebrate or insect models. Their mixed‐mating systems allow natural contrasts between clonality and recombination, shedding light on adaptation and genetic trade‐offs (Doums et al. 1996; Felmy et al. 2023).

From an applied perspective, freshwater snails are sensitive bioindicators of environmental quality, responding rapidly to temperature, salinity, oxygen, or pollution gradients (Chen et al. 2021). In addition, stable isotope signatures preserved in snail shells can provide retrospective information on past environmental conditions, including temperature and precipitation regimes. Their shells record chemical signatures of environmental exposure, and their community composition mirrors habitat integrity (Immenhauser et al. 2016). With modern genomic and environmental DNA (eDNA) tools, it is now possible to simultaneously assess snail diversity, detect parasites, and infer ecological interactions (Garcia‐Moreno et al. 2014; McMahon 1983). Environmental DNA tools in particular enable high‐resolution upscaling of freshwater biodiversity patterns, providing a powerful complement to traditional malacological surveys (Carraro et al. 2020). In parallel, integrative barcoding frameworks developed for snail‐borne trematodes demonstrate how molecular identification of both snails and parasites can operationalize a One Health approach to trematodiases (Schols et al. 2020). More recently, MALDI‐TOF mass spectrometry has emerged as a rapid and field‐relevant tool for identifying schistosome‐transmitting freshwater snails (Hamlili et al. 2021). Finally, genomic resources such as the *Biomphalaria glabrata* reference genome provide new opportunities to dissect immune, stress‐response, and compatibility pathways underlying vector competence (Adema et al. 2017). Yet snails remain peripheral to most One Health and Planetary Health frameworks (Whitmee et al. 2015). Their dual identity as ecosystem engineers and disease vectors situates them at the biodiversity–climate–health interface, but they are rarely recognized as such. Research has long separated ecological questions (e.g., invasion, adaptation, resilience) from health questions (e.g., vectorial competence, disease emergence), even though both operate on the same organisms under the same environmental gradients. This disciplinary divide prevents us from exploring eco‐evolutionary feedbacks—the reciprocal influences of ecological and evolutionary processes—that determine disease transmission and ecosystem functioning (Boots and Sasaki 2002; Day and Gandon 2007).

Re‐centering freshwater snails as integrative models can bridge this gap. Their biology couples environmental sensitivity, reproductive flexibility, and host–parasite interactions, making them perfect sentinels of the freshwater nexus. Recognizing their value expands the conceptual foundations of eco‐evolutionary and One Health research, providing general insights into how adaptation and biodiversity jointly determine ecosystem resilience and disease emergence in a changing world.

## 2 Freshwater Snails as Nexus Sentinels

**FIGURE 1:**
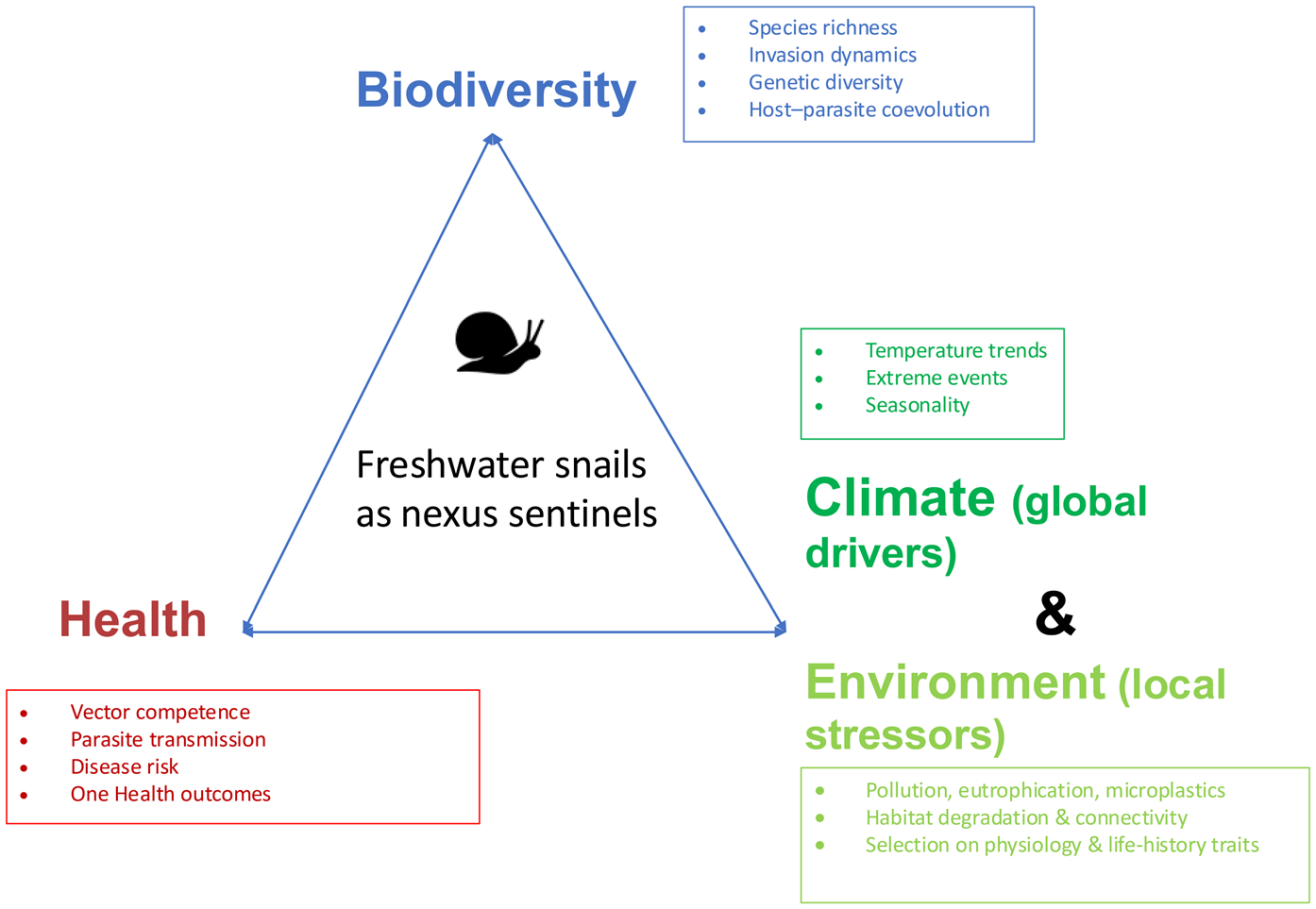
Freshwater snails as “nexus sentinels” integrating biodiversity dynamics, climate drivers, local environmental stressors, and health processes. Climate refers to large‐scale, long‐term drivers, whereas environment refers to local, immediate stressors. The conceptual triangle illustrates how eco‐evolutionary feedbacks and multi‐stressor responses jointly shape parasite transmission and disease risk under global change. All text associated with each driver is enclosed within a single shape for clarity. Combined pressures may generate non‐additive effects that increase the likelihood of disease emergence.

The diversity and dynamics of freshwater snail biology make them ideal to study how biodiversity, climate, and health interact. They lie at the center of a network where environmental stressors, host–parasite relationships, and community dynamics converge. This intersection can be visualized in a conceptual triangle (Figure 1), with vertices representing biodiversity, climate and environment, and health, and snails acting as integrative sentinels connecting all three domains. Although climatic drivers and local environmental variables can influence freshwater systems independently, they are intentionally grouped here to maintain a parsimonious conceptual framework, while their distinct roles are explicitly distinguished in the text.

### 2.1 Biodiversity Dimension: Evolution in Motion

Freshwater snail communities reveal strong contrasts between native and invasive taxa. The family Lymnaeidae, for instance, includes both selfing and outcrossing species, endemic lineages and cosmopolitan invaders (Burgarella et al. 2015; Alda et al. 2021). Selfing species such as *Galba truncatula* show strong spatial structure, limited dispersal, and local adaptation to parasites or stress (Chapuis et al. 2007), whereas invaders like *Physa acuta* or *Pseudosuccinea columella* combine dispersal, plasticity, and rapid evolution (Chapuis et al. 2017, 2024). These natural contrasts allow testing how mating systems and gene flow shape adaptation and invasion success (Jarne et al. 2021).

At community scale, the arrival of invasive hosts can disrupt or amplify transmission cycles. Invasive species may act as dead‐end hosts or as amplifiers, altering infection networks and thereby linking biodiversity change to health outcomes (Johnson et al. 2009; Civitello et al. 2015). Snails thus embody evolution in action, offering tractable systems to quantify selection, drift, and gene flow across climatic gradients and invasion fronts.

### 2.2 Climate and Environment Dimension: Multiple Stressors and Adaptive Responses

Here, I distinguish climate as large‐scale, long‐term drivers (e.g., temperature regimes and extreme events) from local environmental conditions and stressors (e.g., pollution, eutrophication, and habitat degradation), while emphasizing their combined effects on freshwater snail biology. Snails are acutely sensitive to environmental stress. Their survival, growth, and reproduction depend on temperature, hydrology, and water chemistry. Warming accelerates metabolism but reduces survival or size (Deutsch et al. 2008; O'Connor 2025); altered rainfall affects connectivity (Amoros and Bornette 2002; Zheng et al. 2022); eutrophication and microplastics alter physiology and immunity (Weber 2021; Khanjani 2023).

Living at the air–water interface exposes them to sharp thermal gradients, diel hypoxia, contaminants and parasitism, so freshwater snails and mussels provide tractable models for disentangling combined effects of multiple stressors on physiology, behavior, and host–parasite interactions (Ormerod et al. 2010; Zayed et al. 2022; Dai et al. 2025).

Rapid evolution of thermal and pollutant tolerance has been documented within few generations, supported by transcriptomic and quantitative‐genetic evidence (Barrett et al. 2011; Seebacher et al. 2015). Snails can thus inform general principles of adaptation to fluctuating environments, including how phenotypic plasticity and genetic responses interact under multi‐stressor conditions to shape resilience, persistence, and disease risk. Their distributions are also shifting with climate: warming extends transmission windows in temperate zones, while droughts reshape habitats in the tropics (Mas‐Coma et al. 2022). Ecological niche models reconstructing historical distributions of schistosomiasis‐transmitting snails in South Africa further illustrate how temperature and rainfall variability jointly shape snail abundance and range limits (Ayob et al. 2023). Range expansions, such as those observed for *P. columella* (Lounnas et al. 2017) or *G. truncatula* (Alda et al. 2021), illustrate how environmental pressures, phenotypic plasticity, and evolutionary processes interact. Because their distributions integrate environmental signals, snails act as biological recorders of climate stress and predictors of epidemiological change.

### 2.3 Health Dimension: Vectors and Regulators of Disease

Freshwater snails are intermediate hosts for trematodes causing schistosomiasis, fasciolosis, and other water‐borne diseases that affect millions of people (Colley et al. 2014; Hurtrez‐Boussès et al. 2023). Infection outcomes depend on the interplay between snail genotype, parasite strain, microbiota, and environment (Coustau et al. 2015; Portet et al. 2021). Their immune responses—hemocyte activity, lectins, oxidative defenses—offer a window into invertebrate immunity evolution.

Snails also regulate disease risk indirectly. In species‐rich communities, functional redundancy can dilute infection risk, whereas disturbed habitats dominated by tolerant invaders often amplify transmission (Johnson et al. 2009; Keesing et al. 2010; Rohr et al. 2020). They thus act simultaneously as vectors and regulators, connecting ecological integrity with epidemiological stability.

### 2.4 An Integrative Framework

These three dimensions converge in the conceptual triangle of the biodiversity–climate–health nexus. At an ecological scale, snail abundance reflects habitat quality; at an evolutionary scale, their genetic diversity captures adaptive responses; at an epidemiological scale, they mediate parasite transmission while reflecting ecosystem health.

This integrative view enables cross‐scale studies combining field data, genomics, and modeling. Laboratory experiments can measure genetic covariances between stress tolerance, reproduction, and infection outcomes. Field surveys integrating eDNA and infection diagnostics can map co‐responses of snails and parasites to climate gradients. Eco‐epidemiological models incorporating these data can forecast changes in disease risk (Gandon and Day 2009).

By integrating ecology, evolution, and epidemiology within one tractable system, freshwater snails transform the abstract nexus into a measurable framework that links biodiversity change, environmental stress, and health outcomes. Figure 1 synthesizes this integrative framework by explicitly distinguishing climate drivers from local environmental stressors within a single dimension while positioning freshwater snails as central nexus sentinels linking biodiversity and health.

## 3 Toward a One Health Integration of Eco‐Evolutionary Models

Integrating eco‐evolutionary theory with health frameworks is a major challenge for 21st‐century biology. One Health and Planetary Health recognize that human, animal, and environmental health are interdependent (Whitmee et al. 2015; Destoumieux‐Garzón et al. 2018), yet most applications remain descriptive. Recent work in Planetary Health has also emphasized the mental and social dimensions of environmentally mediated disease burdens, thereby broadening nexus thinking beyond strictly physical health outcomes (e.g., Kumar et al. 2023). They seldom incorporate evolutionary and ecological mechanisms that govern disease emergence, persistence, or decline. Importantly, health impacts are often implicitly equated with acute lethality, whereas many environmentally mediated diseases—particularly those transmitted by freshwater mollusks—are primarily characterized by high prevalence, chronic morbidity, and long‐term socio‐economic consequences rather than high case‐fatality rates. Freshwater snails meet the need for systems that are both ecologically relevant and experimentally tractable. Unlike many highly mobile vectors or reservoir hosts (e.g., mosquitoes, ticks, or rodents), freshwater mollusks are tightly coupled to local environmental conditions, making them particularly powerful sentinels for integrating biodiversity, climate, and disease processes.

### 3.1 Bridging Ecological and Health Sciences

Snail systems operationalize One Health principles within an eco‐evolutionary framework. In the field, they respond rapidly to climate and habitat change, providing early warning of degradation. In the laboratory, they permit controlled experiments on adaptation, immunity, and transmission. Their dual identity as ecosystem indicators and parasite hosts allows testing hypotheses across disciplines—how environmental stress modifies immunity, how selfing affects disease persistence, or how invasion alters transmission networks (Boots and Sasaki 2002; Coustau et al. 2015; Day et al. 2020).

This integrative potential contrasts with the reductionist tradition of vector biology, which isolates infection from broader ecological contexts. Considering snails as evolving components of ecosystems reveals feedbacks between biodiversity, adaptation, and pathogen evolution—feedbacks essential to long‐term epidemiological outcomes.

### 3.2 From Local Observations to Global Frameworks

Snails occur on all continents except Antarctica, enabling replicable cross‐climate comparisons (Strong et al. 2008). Contrasts between native and invasive lineages—such as *Physa acuta* , *Galba truncatula* , and *Pseudosuccinea columella* —illustrate how invasion, admixture, and rapid evolution modify vector competence (Lounnas et al. 2017; Alda et al. 2021). These “natural experiments” connect macroecological patterns with microevolutionary processes, grounding predictive models of disease emergence (Gandon and Day 2009; Pedersen et al. 2014; Mari et al. 2017). Transmission models that explicitly incorporate snail population age structure show that host demography substantially alters the relationship between snail and human prevalence, reinforcing the need to integrate realistic snail population dynamics into evolutionary‐epidemiological frameworks (Anderson et al. 2021).

Their simplicity also supports capacity building in endemic regions: snail monitoring, molecular identification, and parasite screening require modest resources, enabling equitable participation in One Health initiatives (Destoumieux‐Garzón et al. 2018).

### 3.3 Rethinking One Health Through an Evolutionary Lens

Evolutionary biology remains underused in health sciences, despite its relevance to pathogen and host dynamics (Boots and Sasaki 2002; Day et al. 2020). Snails demonstrate that evolution acts on epidemiological timescales. Their rapid adaptation to stress and infection shows that host–parasite systems evolve continuously under climate and anthropogenic pressures (Mas‐Coma et al. 2022). Explicitly accounting for these eco‐evolutionary processes in predictive frameworks will improve our capacity to anticipate disease risk.

Snail systems also challenge the false dichotomy between biodiversity and health. Preserving diversity often stabilizes communities and reduces infection, whereas disturbance and invasion homogenize assemblages and amplify disease (Keesing et al. 2010; Rohr et al. 2020). Understanding these patterns requires explicit attention to the evolution of tolerance, resistance, and life‐history traits—insights that snail systems can uniquely provide.

### 3.4 A Call for Integration

Recognizing freshwater snails as model systems for the biodiversity–climate–health nexus opens new directions for both research and policy. Conceptually, they connect eco‐evolutionary theory with applied questions in conservation and health. Practically, they offer cost‐effective, scalable systems for interdisciplinary work.

I propose thus three priorities:

1. Integrate evolutionary and environmental monitoring within One Health programmes, using snails as bioindicators of stress and pathogen circulation.
2. Encourage interdisciplinary networks combining evolutionary ecology, parasitology, and public health.
3. Include freshwater mollusks in global biodiversity and health assessments, alongside traditional vertebrate and insect models (Whitmee et al. 2015; Destoumieux‐Garzón et al. 2018).

These actions align with international calls for early‐warning systems and integrated disease surveillance (Hassan et al. 2023; European Food Safety Authority 2023). Beyond their utility, they symbolize a shift from anthropocentric health paradigms toward genuinely integrative, evolution‐aware frameworks.

### 3.5 Conclusions and Perspectives

Freshwater snails are small and inconspicuous, yet central to the framework linking biodiversity, climate, and health. They embody One Health in action—interdependence among species, environments, and evolutionary processes. Studying them connects molecular mechanisms of adaptation to ecosystem and public‐health outcomes.

Their inclusion in One Health and Planetary Health discourses is not symbolic but operational. Snails ground these frameworks in testable, scalable models that capture the complexity of global change. They remind us that sustainable responses to disease and biodiversity loss must integrate ecology, evolution, and society.

In an era of accelerating climate disruption, biological invasions, and emerging diseases, we need models that are both integrative and tractable. Freshwater snails offer precisely that: living interfaces where environmental change, adaptation, and health outcomes converge. Recognizing them as nexus sentinels will enrich eco‐evolutionary science and strengthen our collective ability to predict and mitigate the impacts of global change on both biodiversity and human well‐being.

## Author Contributions

**Elodie Chapuis:** conceptualization (equal), resources (equal).

## Funding

This work was supported by the Institut de Recherche pour le Développement (IRD), the UMR MIVEGEC (Univ. Montpellier, CNRS, IRD) and by the ANR grant ZOOCAM (ANR‐23‐PEPZ‐0004).

## Conflicts of Interest

The author declares no conflicts of interest.

## Data Availability

No new data were generated or analyzed in support of this research.
